# Extent of piriform cortex resection in children with temporal lobe epilepsy

**DOI:** 10.1002/acn3.51852

**Published:** 2023-07-20

**Authors:** Rory J. Piper, Debayan Dasgupta, Maria H. Eriksson, Mathilde Ripart, Almira Moosa, Aswin Chari, Kiran K. Seunarine, Chris A. Clark, John S. Duncan, David W. Carmichael, Martin M. Tisdall, Torsten Baldeweg

**Affiliations:** ^1^ Developmental Neurosciences Research and Teaching Department UCL Great Ormond Street Institute of Child Health London UK; ^2^ Department of Neurosurgery Great Ormond Street Hospital London UK; ^3^ Department of Clinical and Experimental Epilepsy, UCL Queen Square Institute of Neurology University College London London UK; ^4^ Victor Horsley Department of Neurosurgery National Hospital for Neurology and Neurosurgery London UK; ^5^ Neuropsychology Great Ormond Street Hospital NHS Trust London UK; ^6^ Department of Neurology Great Ormond Street Hospital NHS Trust London UK; ^7^ Department of Biomedical Engineering King's College London London UK

## Abstract

**Objective:**

A greater extent of resection of the temporal portion of the piriform cortex (PC) has been shown to be associated with higher likelihood of seizure freedom in adults undergoing anterior temporal lobe resection (ATLR) for drug‐resistant temporal lobe epilepsy (TLE). There have been no such studies in children, therefore this study aimed to investigate this association in a pediatric cohort.

**Methods:**

A retrospective, neuroimaging cohort study of children with TLE who underwent ATLR between 2012 and 2021 was undertaken. The PC, hippocampal and amygdala volumes were measured on the preoperative and postoperative T1‐weighted MRI. Using these volumes, the extent of resection per region was compared between the seizure‐free and not seizure‐free groups.

**Results:**

In 50 children (median age 9.5 years) there was no significant difference between the extent of resection of the temporal PC in the seizure‐free (median = 50%, *n* = 33/50) versus not seizure‐free (median = 40%, *n* = 17/50) groups (*p* = 0.26). In a sub‐group of 19 with ipsilateral hippocampal atrophy (quantitatively defined by ipsilateral‐to‐contralateral asymmetry), the median extent of temporal PC resection was greater in children who were seizure‐free (53%) versus those not seizure‐free (19%) (*p* = 0.009).

**Interpretation:**

This is the first study demonstrating that, in children with TLE and hippocampal atrophy, more extensive temporal PC resection is associated with a greater chance of seizure freedom—compatible with an adult series in which 85% of patients had hippocampal sclerosis. In a combined group of children with and without hippocampal atrophy, the extent of PC resection was not associated with seizure outcome, suggesting different epileptogenic networks within this cohort.

## Introduction

In children with drug‐resistant focal epilepsy, surgery may be an appropriate therapy and, in carefully selected children, has the potential to deliver either seizure freedom or a meaningful reduction in seizures.^1^ Approximately 60%–70% of children with temporal lobe epilepsy (TLE) undergoing anterior temporal lobe resection (ATLR) achieve seizure freedom following surgery.^2^, ^3^ There remains a need to determine why the remaining ~30% of children continue to have seizures.^4^, ^5^ The answer(s) will be multifactorial, and research is required to understand these reasons in order to (a) inform surgical candidacy; and (b) inform surgical approach.

Recent studies have identified the extent of resection of the piriform cortex (PC) as an important factor associated with seizure freedom following ATLR in adults.^6^, ^7^, ^8^ The PC is primary olfactory cortex and is located at the mesial temporal and basal frontal lobes and spans the endorhinal sulcus.^9^, ^10^ The PC has connections to the mesial temporal lobe structures (hippocampus and amygdala), basal frontal lobe, limbic system, and thalamus^11^ and the PC has been suggested as a key zone of seizure propagation in patients with TLE.^12^ Prior cohort studies showed that extent of PC resection or ablation was associated with a greater rate of seizure freedom following ATLR.^6^, ^7^, ^8^ In Galovic et al.'s study of 107 adults with mesial TLE undergoing ATLR, the median resection of the whole PC (temporal and frontal parts) was 83% in those seizure‐free versus 52% in those not seizure‐free.^7^ In Borger et al.'s study of 82 adults with mesial TLE undergoing selective amygdalo‐hippocampectomy, the median resection of the temporal part of the PC was 51% in those seizure‐free versus 13% in those not seizure‐free.^8^ Neither study detected a relationship between the extent of resection of the hippocampus or amygdala with seizure freedom, which have previously and conventionally been the surgical targets in performing an ATLR.^13^, ^14^ These studies raise the question as to whether targeted resection of the PC will deliver a higher likelihood of seizure freedom.

No studies have investigated the importance of PC resection in ATLR in children. Pediatric epilepsy surgery cohorts are a more heterogeneous group, with a smaller contingent of hippocampal sclerosis than adult cohorts.^15^ Furthermore, duration of disease is shorter in children than in adults. There is therefore a need to determine whether resection of the PC is as pertinent in children as has been suggested in adults, and in children with different pathologies.

The primary objective of this study was to investigate the relationship between the extent of resection of the temporal PC and postoperative seizure freedom in all children in a TLE cohort undergoing ATLR and specifically in a subgroup with hippocampal atrophy—a group more in‐keeping with the patients in the prior adult TLE cohort studies.^7^, ^8^ The secondary outcomes of the study were to investigate the association between seizure freedom with the extent of resection of the amygdala and hippocampus.

## Methods

### Design

A single‐center, retrospective neuroimaging cohort study was performed. Approval was given by the UCL Great Ormond Street Institute of Child Health Research and Development Department and the Great Ormond Street Hospital Caldicott Guardian (ID = 20NP04). According to local governance approvals, written consent from the patients or their parent(s)/guardian(s) was not required.

### Patients

**Figure 1:**
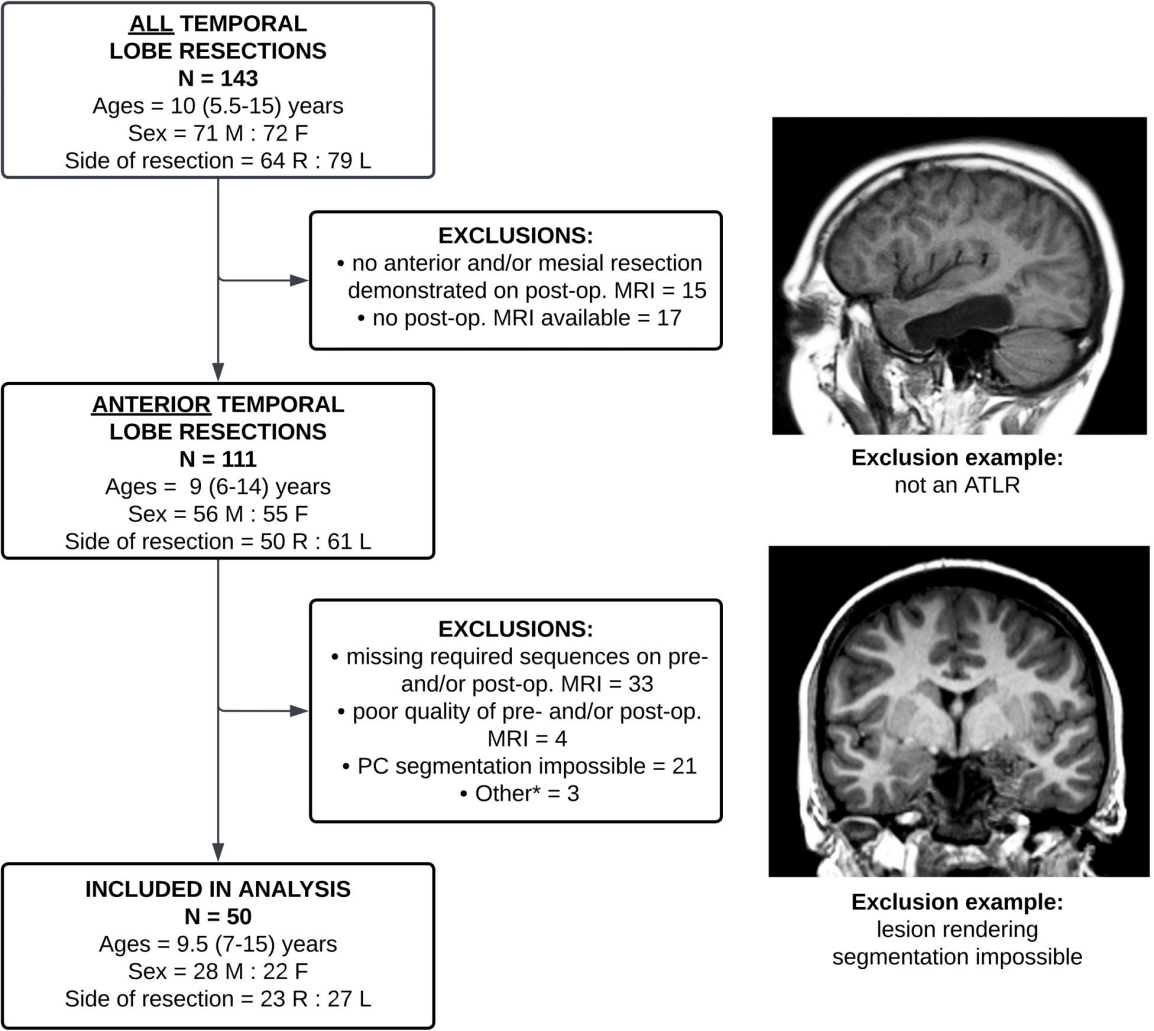
Flowchart demonstrating the initial cohort of children who underwent any form of temporal lobe resection, number of exclusions per category, and the final cohort of children who had necessary data for the intended analysis. *Three children were excluded from the analysis since postoperatively they were found to have evidence of multifocal‐onset epilepsy. Ages are given as a median (and interquartile range). ATLR, anterior temporal lobe resection; F, female; L, left; M, male; R, right.

The operative database at Great Ormond Street Hospital was searched for children who had undergone ATLR between 1st January 2012 and 4th February 2021 (Fig. 1). Prior to 2012, very few patients had undergone MRI scanning with the sequences required for this study, which are described in the following sections. Children with documented seizure outcomes at least one year after surgery were included.

### Clinical data

Clinical documentation was reviewed, and the following data variables were extracted: age, sex, duration of epilepsy, date of surgery, dates of preoperative and postoperative MRIs used for analysis, radiology report, histopathological report, semiology, prior intracranial electroencephalography findings, binary seizure freedom outcome at 1‐year following surgery (seizure‐free or not seizure‐free), and binary seizure freedom outcome at last follow‐up. Seizure freedom was considered as no seizures and no auras (equivalent to an Engel 1A/ILAE 1 outcome).^16^, ^17^

### Image acquisition

MRI studies were extracted from the Great Ormond Street Hospital secure imaging server. Given that this study was retrospective and used existing clinical data, it was not possible to standardize the specific imaging protocols used and these included imaging acquired using both 1.5 Tesla and 3 Tesla clinical MRI scanners. MRI scans were performed by either our institution or by the referring hospitals. The most recent preoperative MRI and the first available postoperative scan were selected, provided they included a volumetric 1 mm^3^ T1‐weighted (T1W) MRI sequence (without intravenous contrast). If these were not available, the next closest MRI study was selected. For those patients who only had a T1W sequence of higher resolution, the images were down‐sampled to 1 mm isotropic spatial resolution.

### Image processing and analysis

**Figure 2:**
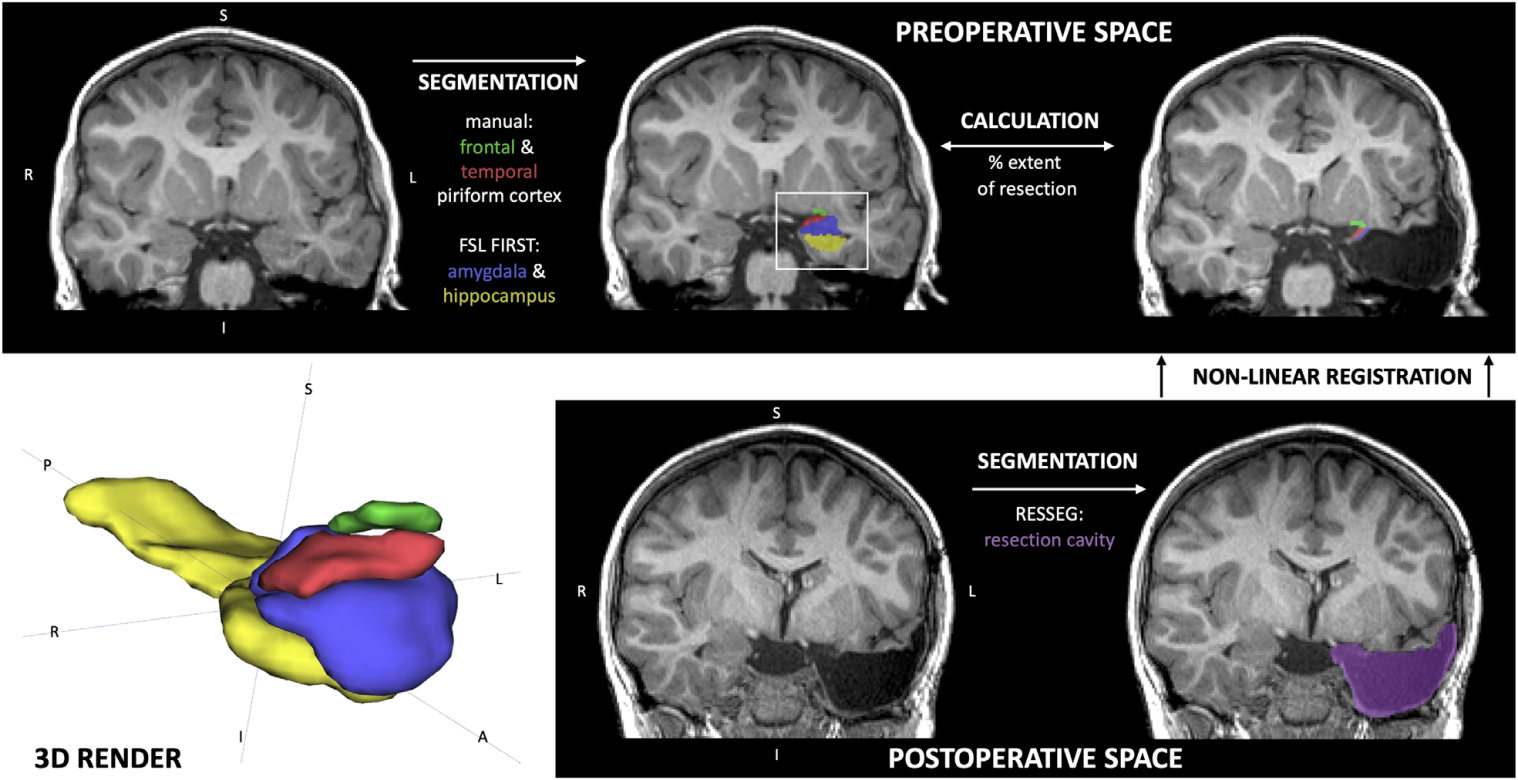
Image processing pipeline in one child who underwent a left anterior temporal lobe resection.

The image processing steps for this study are summarized in Figure 2. The axial plane of the preoperative T1W images were aligned to the long axis of the body of the hippocampus, as per Galovic et al.'s study.^7^ The PC was then manually segmented on both sides, including a sub‐parcellation of the frontal and temporal components.^7^ Manual segmentation was performed by a researcher blinded to seizure freedom outcomes. The right and left amygdala and hippocampi were automatically segmented using FSL FIRST,^18^ and were manually corrected if errors were noted. The resection cavity was automatically segmented on the postoperative T1W images using the RESSEG tool^19^ and then manually corrected. The postoperative T1W images were registered to the preoperative T1W images using a nonlinear registration tool by NiftyReg (“*Reg f3d*”; http://cmictig.cs.ucl.ac.uk/wiki/index.php/Reg_f3d) that used the cavity segmentation as an image mask. FSLMATHS was used to create the resection masks by selecting the overlapping voxels in the cavity mask and the gray and white matter masks generated by Geodesic Information Flow (GIF) parcellation (http://niftyweb.cs.ucl.ac.uk/).^20^ The residual PC was manually segmented on the co‐registered postoperative T1W images. FSLMATHS was used to subtract the resected portions (determined by resection and preoperative label overlaps) of amygdala and hippocampus.^21^ All segmentations were checked and corrected manually. The intracranial volume (ICV) used for ROI volume correction was calculated using the brain extraction tool (“bet”) in FSL.^21^

### Hippocampal atrophy

The patients with hippocampal atrophy were determined quantitatively by calculating the ratio of the ipsilateral to contralateral hippocampal volumes measured on MRI and assigned z‐scores according to a distribution of ratios in a cohort of healthy childhood controls. Rather than z‐scoring each raw hippocampal volume to controls, asymmetry was used to account for intersubject differences in intracranial volume. Seventy‐four children without neurological disorders were scanned with a 1‐mm isotropic MPRAGE sequence with MRI at 3 T. Automated segmentation of the hippocampi was performed using FSL FIRST^18^ and volumes were calculated. The ratio between the right and left was performed after randomizing each control to a right‐to‐left or left‐to‐right ratio. Four children were excluded as the automated segmentation failed. The final group of 70 healthy children had a median age of 12 (range 7–18) years and 55 out of 70 were female. The mean hippocampal ratio was 1.01 (standard deviation ±0.12).

Patients in the TLE cohort were defined as having hippocampal atrophy if they were at least one standard deviation from the mean in the control group. Like the controls, if the FIRST automated segmentation failed (*n* = 4), they were not included in the hippocampal atrophy group.

### Statistical analysis

The volumes of all ROIs were calculated using the MATLAB “*niftiread”* function (Version R2022a). Volumes were controlled for intracranial volume (ROI/intracranial volume) but not for the extent‐of‐resection calculations.

The primary outcome measure was the extent of the resection of the temporal part of the PC.^8^ Extent of resection was calculated as a percentage by the following equation: ([Preoperative ROI—Postoperative ROI]/Preoperative ROI) × 100. Negative extent of resection values were excluded as these are impossible and likely due to segmentation variance—detailed in the inter‐rater analysis. The differences in extent of resection between the seizure‐free and non‐seizure‐free groups were analyzed using an unpaired Wilcoxon test. Ipsilateral and contralateral ROIs were compared using a paired Wilcoxon test. Demographic and categorical data were compared using Fisher's tests or chi‐squared test. A Bland–Altman plot was used for analysis and demonstration of the agreement in PC measurement (including the frontal and temporal part) between two independent raters.

Statistical analyses were conducted in R Studio Version (V4.1.0). Statistical tests used *p <* 0.05 as statistically significant. Months, years, and percentages are rounded to the nearest integers.

## Results

### Patients and clinical characteristics

A total of 143 children who underwent temporal lobe resection (including, but limited to, ATLR) at Great Ormond Street Hospital (London, UK) between 1st January 2012 and 4th February 2021 were identified. Thirty‐two were excluded as either the postoperative MRI did not demonstrate an ATLR (*n* = 15) or there was no postoperative MRI to verify the resection (*n* = 17). Other reasons for exclusion were missing sequences on either the preoperative or postoperative MRI scan (*n* = 33), impossibility of PC segmentation due to deforming lesions, and/or motion artifact (*n* = 21) or poor quality of the MRI(s) (*n* = 4) (Fig. 1). Three children were excluded from the analysis since postoperatively they were found to have evidence of multifocal‐onset epilepsy.

The remaining cohort included 50 children (22 female, 27 left sided) with a median age at the time of surgery of 9.5 years (IQR = 7–15 years) (Table 1). Thirty‐three were seizure‐free at final follow‐up following surgery and the median final follow‐up was 22 months (IQR = 15–44 months). The median duration between surgery and postoperative MRI was 6 months (IQR = 4–9 months). Tumors (19 out of 50) and hippocampal sclerosis (19 out of 50) were the most frequent primary pathologies reported, followed by focal cortical dysplasia (6 out of 50). Two of the 50 children had a documented preoperative olfactory and/or gustatory aura. Seven of the 50 children were investigated with intracranial (stereotactic) electroencephalography prior to ATLR. One of the seven children had a seizure‐onset network involving both the temporal and ipsilateral cingulate regions—this child was not seizure‐free postoperatively. A case‐by‐case description of the cohort is provided in Table S1.

**Table 1:** Clinical variables of the patient cohort. All ages, months, years, and percentages are rounded to nearest integer.

Variables	Total TLE group	Seizure‐free	Not seizure‐free	*p*‐value	TLE group with hippocampal atrophy	Seizure‐free	Not seizure‐free	*p*‐value
Number of patients	50	33	17	–	19	13	6	–
Sex								
M	28	14	14	–	9	4	5	–
F	22	19	3		10	9	1	
Age at surgery (median [IQR]) (years)	9.5 (7–15)	11 (7–15)	8 (6–15)	0.59	11 (8–15)	13 (9–16)	8 (7–9)	0.06
Duration of epilepsy (median [IQR]) (years)	6 (4–11)	6 (5 – 11)	6 (4–9)	0.98	7 (5–10)	9 (5–12)	5 (4–7)	0.10
Number of patients with focal‐to‐bilateral tonic–clonic seizures	23	13	10	0.31	8	5	3	1
Follow‐up duration (median [IQR]) (months)	22 (15–44)	18 (14–39)	25 (19–44)	.09	25 (16–46)	18 (15–40)	39 (27–69)	0.09
Side of surgery								
R	23	16	7	0.77	9	6	3	1
L	27	17	10		10	7	3	
Pathology								
Tumor	20	16	4	0.05	4	3	1	0.16
HS only	19	11	8		13	10	3	
FCD	6	5	1		1	0	1	
Other	5	1	4		1	0	1	
Total volume of temporal lobe resection (mm^3^), corrected for intracranial volume (mm^3^) (median [IQR])	0.023 (0.017– 0.027)	0.022 (0.016– 0.027)	0.023 (0.021– 0.026)	0.36	0.022 (0.019–0.028)	0.023 (0.019– 0.029)	0.020 (0.019–0.025)	0.42
Extent of resection (median [IQR]) (%)								
PC temporal	46 (19–72)	50 (13–77)	40 (21–63)	0.26	43 (19–76)	53 (43–77)	19 (7–32)	0.009*
PC frontal	0 (0–8)	0 (0–9)	0 (0–4)	0.75	0 (0–2)	0 (0–2)	0 (0–1)	0.88
Hippocampus	70 (55–80)	64 (22–80)	73 (70–79)	0.08	76 (70–85)	79 (70–86)	71 (60–73)	0.13
Amygdala	78 (60–90)	75 (58–89)	81 (72–94)	0.38	84 (69–92)	86 (72–90)	80 (64–91)	0.83

Demographic and categorical data were compared using Fisher's tests for categorical data (other than the analysis for the focal‐to‐bilateral tonic–clonic seizures, which used a chi‐squared test), else the Wilcoxon test was used.

F, female; FCD, focal cortical dysplasia; HS, hippocampal sclerosis; IQR, interquartile range; L, left; M, male; PC, piriform cortex; R, right.

* Marks statistical significance between the seizure‐free and not seizure‐free groups.

We investigated a subgroup of 19 children who had hippocampal atrophy determined quantitatively by hippocampal asymmetry. The demographics and clinical data for these children are also summarized in Table 1. Thirteen out of 19 children with hippocampal atrophy had a histopathological diagnosis of hippocampal sclerosis alone, the remaining seven children had a co‐pathology (four had a tumor, one had a focal cortical dysplasia [Type 3], and one had tuberous sclerosis complex [Type 1]).

### Extent of resection

**Figure 3:**
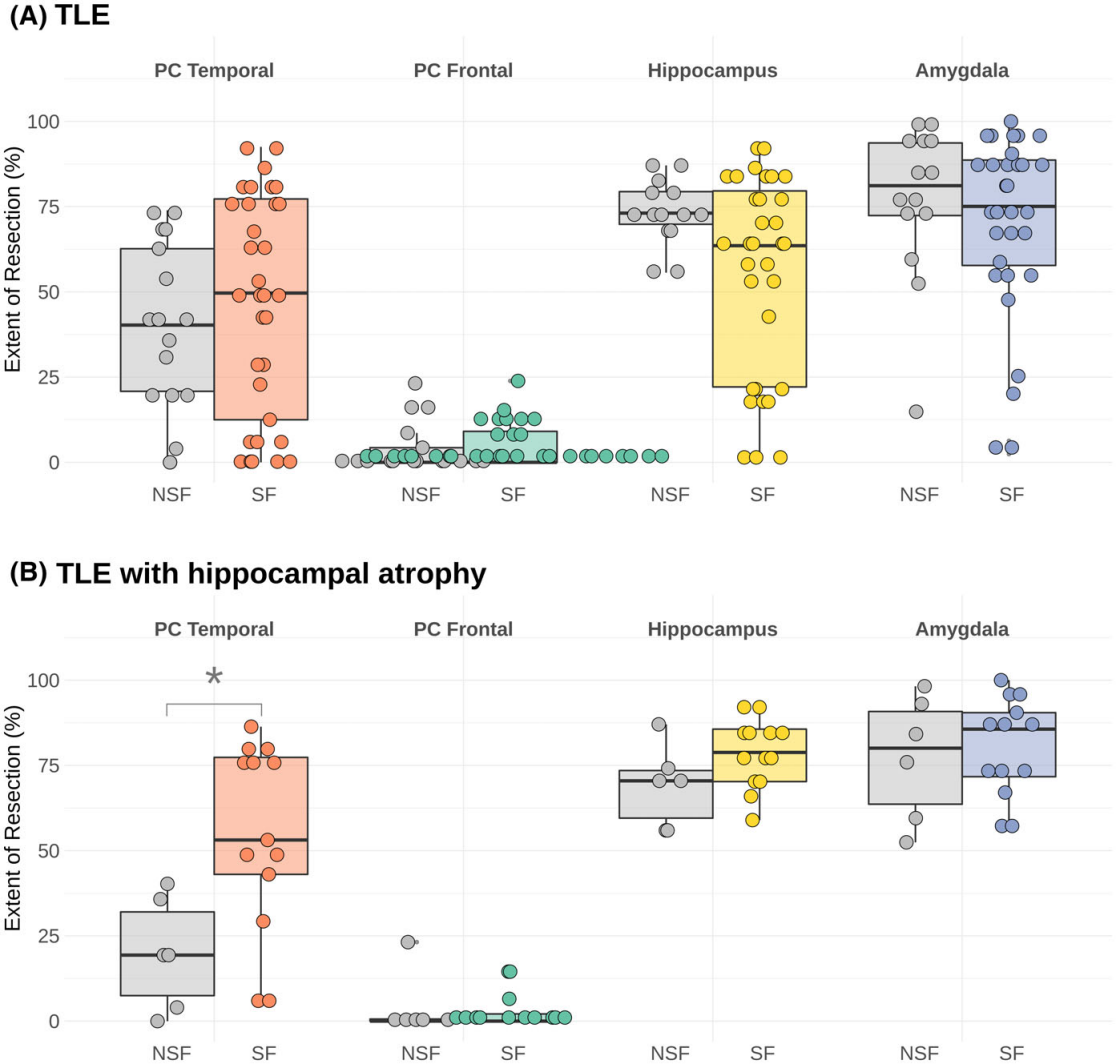
Boxplots showing the relationship between extent of resection of the piriform cortex (PC) (temporal and frontal parts), hippocampus and amygdala in (A) all children with TLE and (B) those children with TLE and hippocampal atrophy. *Shows statistical significance (*p* = 0.009). The colored boxplots show the seizure‐free (SF) group for each region and the gray boxplots the respective not seizure‐free (NSF) group.

For the total cohort, the median extent of resection of the temporal PC in the seizure‐free group was 50% (IQR = 13%–77%) versus 40% (IQR = 21%–63%) in the not seizure‐free group (*p* = 0.26) (Fig. 3). In the children with TLE and hippocampal atrophy, the extent of PC resection was greater (median = 53%, IQR = 43%–77%) than in those who were not seizure‐free group (median = 19%, IQR = 7%–32%) (*p* = 0.009). There were no significant differences in the extent of resection of the frontal PC, amygdala, or hippocampus, nor were there differences in the corrected resection cavity volumes or duration of epilepsy between the seizure‐free and non‐seizure‐free groups in either the total TLE cohort or those children with hippocampal atrophy only.

### Preoperative volumes

**Figure 4:**
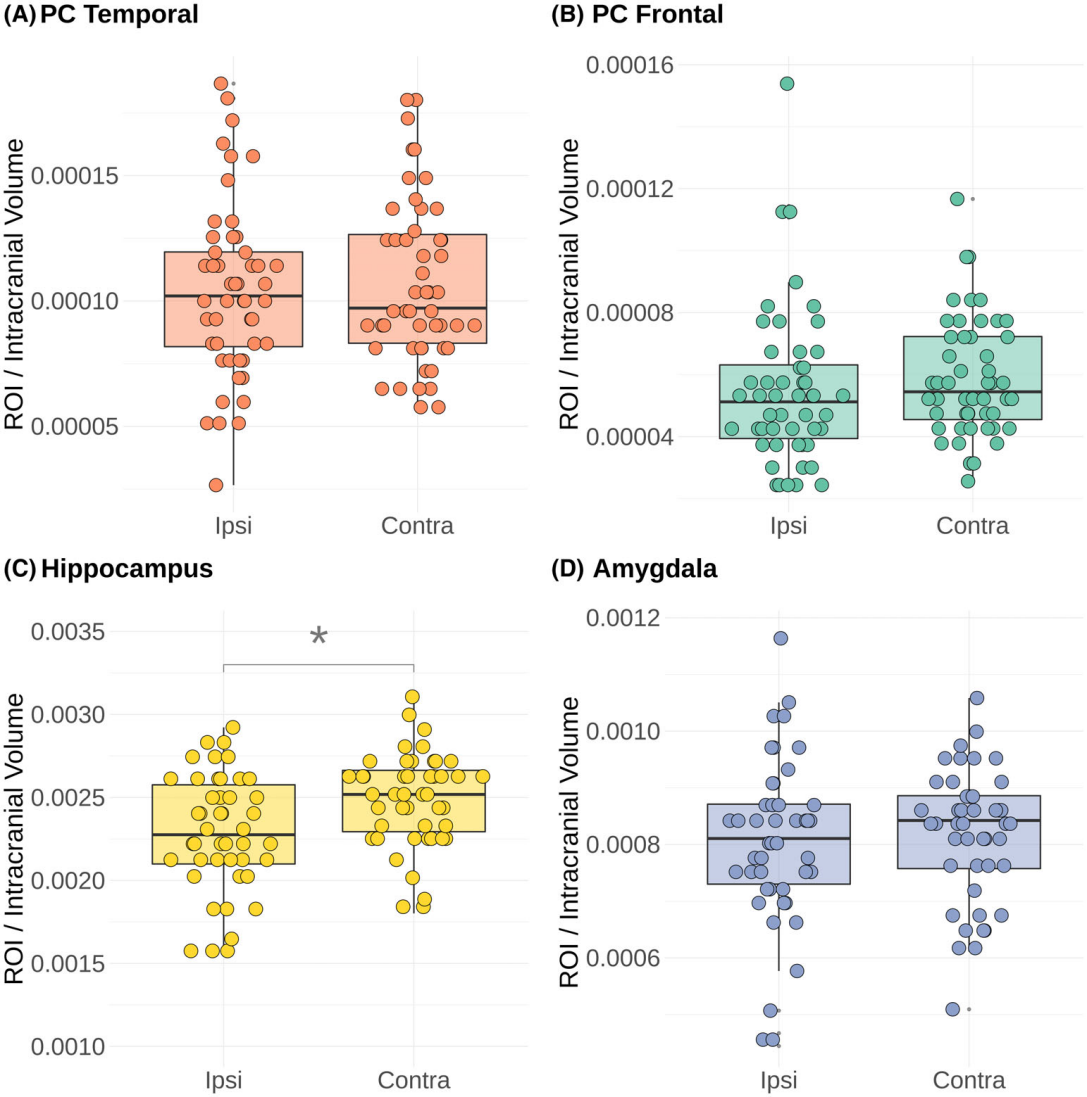
Preoperative volumes for the (A) temporal part of the piriform cortex (PC), (B) frontal part of the PC, (C) amygdala, and (D) hippocampus in the entire TLE cohort (*n* = 50). The preoperative and postoperative PC ROIs could be parcellated in all 50 children, but in 5 out of 50 and 4 out of 50 parcellations of the amygdala and hippocampus could not be performed, respectively, due to lesions in these regions. There were no significant differences in the volumes of the piriform cortex (temporal or frontal parts) or amygdala between the ipsilateral and contralateral sides. The median hippocampal volume was smaller on the ipsilateral than contralateral side. *Shows statistical significance (*p* = 0.0035). Marks statistical significance in the differences detected between the ipsilateral (“Ipsi”) and contralateral (“Contra”) sides to the resection.

There were no significant differences in the preoperative volumes of the temporal or frontal parts of the PC or the amygdala between the ipsilateral (side of resection) and contralateral sides. The ipsilateral hippocampal volume was smaller than the contralateral hippocampal volume (Fig. 4). In the hippocampal atrophy group, there were no differences in the preoperative volumes of the temporal or frontal parts of the PC or the amygdala between the ipsilateral and contralateral sides.

### Inter‐rater analysis

**Figure 5:**
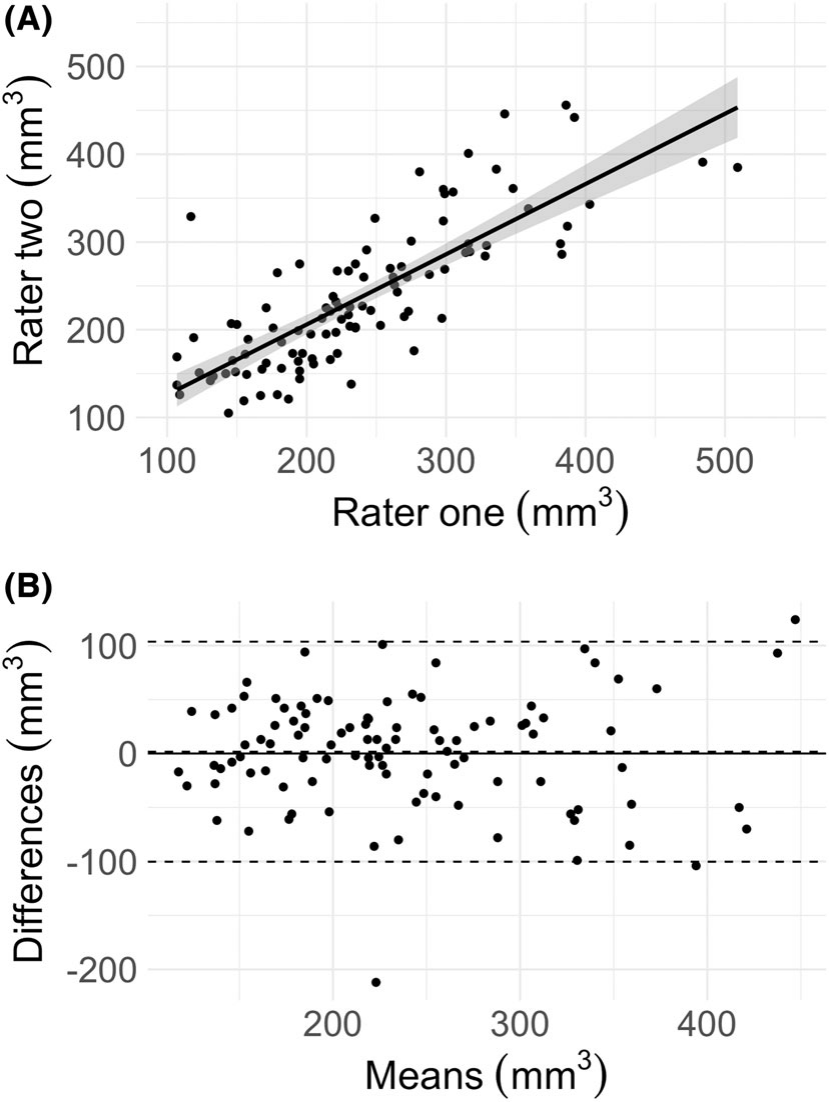
Inter‐rater agreement for segmentation of the piriform cortex volumes (mm^3^) (including the frontal and temporal parts) between two raters.

Figure 5A shows a scatter plot of the volumes (mm^3^) segmented by rater one (x‐axis) versus those segmented in the same cases by rater two (y‐axis). Figure 5B shows the Bland–Altman plot demonstrating the differences between rater one and rater two as shown in Figure 5A. The Bland–Altman analysis showed an acceptable bias of 1.67, an upper limit of agreement of 103.59 mm^3^, and a lower limit of agreement of −100.25 mm^3^.

## Discussion

The objective of this study was to investigate the relationship between the extent of resection of the temporal PC and postoperative seizure freedom in children with TLE who underwent ATLR. In children with TLE and hippocampal atrophy, resection of the temporal portion of PC was higher in those seizure‐free than those not seizure‐free postoperatively (53% vs. 19%, respectively). This finding is consistent with other studies of adults with mesial TLE,^6^, ^7^, ^8^ in which 82%–85% of individuals had hippocampal atrophy. This is the first report of this association in children with TLE. Finding this association between the extent of resection of the PC and postoperative seizure freedom in children as previously found adults is critical from a mechanistic point of view, as the much shorter duration of epilepsy in children compared to adult samples negates the idea that the significance of the piriform cortex is related to the chronicity of the disease.

This association was not, however, found in a wider and more heterogeneous pediatric TLE cohort. This is an important finding as it suggests that not all children with TLE would necessarily benefit from extensive PC resection in ATLR. We hypothesized that children with hippocampal atrophy would be a group that more closely resembles the prior adult cohorts with mesial TLE (~40% of this cohort had hippocampal sclerosis vs. the 85% in Galovic's cohort)^7^ and would also show a relationship between the extent of PC resection and seizure freedom. This makes conceptual sense due to the proximity of the PC to the mesial temporal lobe structures and circuit of Papez.^22^ The thesis that the PC is a key component of epileptogenic networks in mesial TLE is supported by our results. There was no relationship found between the extent of resection of the hippocampus or amygdala with seizure outcome, matching findings from prior studies in adults.^6^, ^7^, ^8^

Our results show an association between seizure freedom and the extent of resection of the temporal part of the PC. This is consistent with Borger et al.'s study in adults.^8^ This is an important distinction considering that the frontal part of the PC is not a region that is amenable to resection given its proximity to the anterior perforating substance and to perforating vessels to the deep subcortical brain structures. As evidenced by this and other studies, resection of the temporal part of the PC has been shown to be possible and gives feasibility to prospective studies of temporal PC resection. However, while there is an ongoing prospective clinical trial of PC resection in adults,^23^ further verification is needed to understand the role of the PC in children with TLE and to identify those children for whom a tailored PC resection is indicated.

This study did not detect a difference between the preoperative volumes of the ipsilateral (side of resection) and contralateral PC. This is different to Galovic et al.'s study, which found that the PC was smaller on the ipsilateral side.^7^ Iqbal et al. showed that the volume of the PC is related to the duration of epilepsy.^24^ This may explain in part the differences between the results in children and adults, as the former had a shorter duration of epilepsy.

This study has several limitations. First, the sample size in this study was small due to the limited availability of isotropic neuroimaging sequences. A small effect size of the relationship between PC extent of resection and postoperative seizure freedom may indeed exist in the overall pediatric TLE cohort, but the large heterogeneity in this relatively small sample may have masked this. We intend to collaborate with other children's epilepsy surgery centers to conduct a larger and external validation study to confirm these results. Second, although we investigated a consecutive cohort, data were not available for all patients, which may present unintentional bias in the study results. Approximately two‐thirds of the consecutive sample were excluded due to MRI data unavailability, poor MRI data quality, or lesions rendering segmentation impossible. Third, although we used hippocampal asymmetry (ipsilateral‐to‐contralateral ratio of hippocampal volumes) to control for intracranial volumes, our method may have been inaccurate in cases of bilateral hippocampal sclerosis. Fourth, manual segmentation of the PC is challenging, particularly in the presence of tumors and other lesions that distort the normal anatomy of the mesial temporal lobe. This is reflected in our inter‐rater analysis that shows variance of approximately ±100 mm^3^ within 95% confidence intervals in the PC volumes measured by two independent raters (Fig. 5). Finally, as this was a retrospective and observational study, the images available were several months following surgery and the resection volumes measured may be confounded by atrophy related to Wallerian degeneration.

The exact anatomical boundaries of the PC remain contentious and recent studies have sought to refine these. For example, a study by Ding et al. examined postmortem brains to identify that a cortical region in the mesial part of the previously named frontal piriform cortex has distinct histological features (different laminar structure and stronger neurofilament protein immunoreactivity) compared to the PC.^25^ The name suggested for this mesial and distinct frontal part of the PC is the “lateral olfactory area.” Manual segmentation of the PC is therefore only as accurate as the segmentation protocol followed and we remain to require further research to refine anatomical accuracy. Furthermore, manual segmentation of the PC is also time consuming and while there is emerging work to demonstrate that the PC can be segmented automatically,^24^, ^26^ a publicly accessible and pediatric‐validated tool is not yet available.

Although several studies suggest that the extent of PC resection is important in achieving seizure freedom in patients with mesial TLE, the role of the PC in epileptogenic and seizure propagation networks is not fully defined.^12^, ^22^ This study was not able to determine if the subgroup of children with hippocampal atrophy have an abnormal mesial temporal network and so further studies are required to underpin the network properties of the PC in TLE. Investigation is limited by the resolution achievable with current clinically available MRI imaging in this region and the complexity of stereotactic electroencephalography recording in this zone where perforating vessels pose a high clinical risk. The application of 7‐Tesla MRI that allows the acquisition of sub‐millimetric structural and functional data may provide an opportunity for further study.^27^, ^28^

## Conclusions

This is the first study that suggests that a greater extent of resection of the temporal portion of PC is associated with a greater chance of seizure freedom in children with TLE and hippocampal atrophy. However, in a cohort of children with TLE with or without hippocampal atrophy, this association between the extent of resection of the temporal PC and postoperative seizure freedom was not found. While a clinical trial in adults is currently underway that aims to prospectively investigate if the purposeful resection of PC during ATLR will deliver greater seizure freedom rates,^23^ further investigation in pediatric cohorts is required before prospective application.

## Author Contributions

RJP, DD, AC, JSD, DWC, MMT, and TB contributed to the conception and design of the study. RJP, MHE, MR, KKS, and CC contributed to acquisition and analysis of the data. RJP, DD, MHE, MR, AC, KKS, CC, JSD, DWC, MMT, and TB contributed to the drafting the manuscript and/or figures.

## Conflicts of Interest

The authors have no conflict of interest to declare.

## Supporting information


Table S1

Appendix S1

## Data Availability

An anonymized summary of each patient's clinical variables is shared in a table as a supplementary file. To preserve the anonymity of the patients whose data are included in this study, we are not able to publish or share the imaging data.
