# Supplementary material for: Extent of piriform cortex resection in children with temporal lobe epilepsy
Source: Ann Clin Transl Neurol. 2023 Jul 20;10(9):1613–22. doi: 10.1002/acn3.51852 (PMC10502684; doi:10.1002/acn3.51852)
Supplement: Supplementary file 1 — Table S1 [file ACN3-10-1613-s002.docx]

**Supplementary Table 1:** Case-by-case description of the patient cohort. M = male; F = female; DNET = dysembryoplastic neuroepithelial tumor; FCD = focal cortical dysplasia; HS = hippocampal sclerosis; MTS = mesial temporal sclerosis; SEEG = stereotactic electroencephalography; SF = seizure-free; NSF = not seizure-free. NA = not applicable (the hippocampi volume ratio could not be calculated in children for whom automated hippocampal volumetry failed). *Marks the children with a hippocampi ratio more than one standard deviation away from the mean of the control cohort.

| **Patient** | **Sex** | **Age at surgery**  (years) | **Duration of epilepsy**  (years) | **Radiological diagnosis / report** | **Lesion(s) location on imaging** | **Histopathology** | **Seizure onset zone hypothesized on SEEG data** | **Seizure freedom (**at one-year) | **Seizure freedom** (at last follow-up) | **Number of post-operative months at last follow-up** (months) | **Hippocampi volume ratio** (ipsilateral : contralateral) |
| --- | --- | --- | --- | --- | --- | --- | --- | --- | --- | --- | --- |
| 1 | M | 17 | 15 | MTS | Mesial | HS | - | SF | SF | 14 | 0.88* |
| 2 | F | 16 | 5 | HS | Mesial | Glioneuronal tumor | - | SF | SF | 15 | 0.79* |
| 3 | M | 15 | 5 | Glioneuronal tumor | Mesial (amygdala) | Glioneuronal tumor | - | SF | SF | 13 | 1.11 |
| 4 | F | 16 | 9 | MTS | Mesial | HS | Hippocampus | SF | SF | 14 | 0.67* |
| 5 | M | 15 | 9 | No lesion identified | No lesion identified | Non-diagnostic changes | Mesial and lateral temporal regions, and cingulate gyrus | NSF | NSF | 16 | n/a |
| 6 | M | 5 | 5 | FCD (grey-white blurring) and HS | Mesial | HS | - | SF | NSF | 15 | 1.00 |
| 7 | F | 15 | 14 | Glioneuronal tumor | Anterior-Mesial (amygdala) | Ganglioglioma | - | SF | SF | 13 | 0.92 |
| 8 | M | 17 | 17 | Glioneuronal tumor | Mesial (hippocampus and fusiform gyrus) | Glioneuronal tumor | - | NSF | NSF | 14 | 0.94 |
| 9 | F | 14 | 14 | Meningoangiomatosis or glioneuronal tumor | Throughout temporal lobe | Glioneuronal tumor | - | SF | SF | 13 | 0.96 |
| 10 | M | 11 | 11 | MTS | Mesial | HS | Hippocampus | SF | SF | 26 | 0.51* |
| 11 | M | 8 | 8 | No lesion identified | No lesion identified | FCD type 3a (inc. HS) | - | SF | NSF | 33 | 0.79* |
| 12 | M | 4 | 4 | Glioneuronal tumor | Mesial (amygdala) | Ganglioglioma | - | SF | SF | 29 | 1.09 |
| 13 | F | 13 | 4 | MTS & anterior grey-white blurring | Mesial | HS | - | SF | SF | 47 | 0.84* |
| 14 | M | 9 | 7 | No lesion identified | No lesion identified | HS | - | SF | SF | 88 | 0.92 |
| 15 | F | 15 | 13 | FCD | Anterior with poorly defined posterior border | FCD type 2b | - | SF | SF | 13 | 1.15 |
| 16 | F | 7 | 6 | Tumor | Mesial | Ganglioglioma & HS | - | SF | SF | 32 | 0.99 |
| 17 | M | 1 | 1 | Glioneuronal tumor | Mesial (amygdala) | Glioneuronal tumor | - | SF | SF | 13 | 1.06 |
| 18 | M | 15 | 15 | Tumor | Mesial | Glioneuronal tumor | - | NSF | NSF | 40 | 1.01 |
| 19 | F | 9 | 7 | Bilateral MTS | Mesial | HS | - | SF | SF | 18 | 0.85* |
| 20 | F | 7 | 6 | MTS | Mesial | HS | - | NSF | NSF | 78 | 0.50* |
| 21 | F | 7 | 5 | HS | Mesial | HS | - | SF | SF | 53 | 0.81* |
| 22 | M | 17 | 8 | HS | Mesial | Hippocampal gliosis | Anterior hippocampus | NSF | NSF | 19 | 1.00 |
| 23 | M | 6 | 6 | FCD | Anterior temporal lobe | Nodular vacuolated neuronal tumor | - | SF | SF | 78 | 0.99 |
| 24 | M | 9 | 8 | MTS | Mesial | HS | - | SF | NSF | 25 | 0.84* |
| 25 | M | 16 | 9 | MTS | Mesial | HS | - | SF | SF | 17 | 0.57* |
| 26 | F | 14 | 13 | MTS | Mesial | HS | - | SF | SF | 30 | 0.88* |
| 27 | M | 12 | 4 | MTS | Mesial | HS | - | SF | NSF | 77 | 0.77* |
| 28 | M | 3 | 2 | Glioneuronal tumor | Anterio-mesial | Ganglioglioma & HS | - | NSF | NSF | 62 | n/a |
| 29 | M | 6 | 6 | FCD or HS | Antero-mesial and superior temporal gyrus | HS | - | NSF | NSF | 63 | 1.02 |
| 30 | M | 18 | 16 | MTS | Mesial (HS) and polar grey-white blurring | HS | - | NSF | NSF | 22 | 0.90 |
| 31 | M | 16 | 6 | FCD | Anterior | FCD type 2b | - | SF | SF | 16 | 1.04 |
| 32 | M | 12 | 11 | FCD or low-grade tumor | Mesial (hippocampus) | HS | - | NSF | NSF | 42 | 1.03 |
| 33 | F | 7 | 1 | Glioneuronal tumor or meningoangiomatosis | Mesial | Glioneuronal tumor | - | SF | SF | 50 | 0.86* |
| 34 | F | 2 | 2 | FCD or MTS or HS | Mesial | Non-specific epilepsy changes | - | NSF | NSF | 19 | n/a |
| 35 | F | 3 | 2 | DNET or FCD | Middle, inferior and parahippocampal gyri. | DNET | - | SF | SF | 45 | 1.14 |
| 36 | F | 15 | 11 | HS | Mesial | FCD type 3 (inc. HS) | - | SF | SF | 18 | 1.04 |
| 37 | F | 7 | 6 | HS | Mesial | HS | - | NSF | NSF | 22 | 0.93 |
| 38 | M | 10 | 4 | DNET | Mesial | DNET | - | SF | SF | 57 | n/a |
| 39 | F | 5 | 5 | MTS | Mesial | HS | - | SF | SF | 14 | 0.88* |
| 40 | M | 15 | 11 | Loss of grey-white border | Diffuse anterior | FCD type 2 | - | NSF | SF | 24 | 0.98 |
| 41 | F | 16 | 5 | DNET | Inferior-lateral | DNET | - | SF | SF | 14 | 1.00 |
| 42 | M | 5 | 4 | Multiple tubers | Bilateral temporal (multiple), left parietal and left occipital | Tuber | Multiple temporal tubers | NSF | NSF | 20 | 0.87* |
| 43 | F | 4 | 3 | Cavernoma | Mesial (amygdala) | Ganglioglioma | - | SF | SF | 47 | 1.06 |
| 44 | M | 8 | 4 | Glioneuronal tumor or FCD | Mesial occipitotemporal and parahippocampal gyri | Glioneuronal tumor | - | NSF | NSF | 44 | 0.81* |
| 45 | F | 17 | 16 | MTS | Mesial | HS | - | SF | SF | 16 | 0.82* |
| 46 | M | 8 | 8 | No lesion | N/A | Non-diagnostic changes | Temporal lobe. | SF | SF | 13 | 1.03 |
| 47 | M | 12 | 12 | Ill-defined lesion | Diffuse | DNET | - | SF | SF | 39 | 0.74* |
| 48 | F | 8 | 8 | FCD | Anterior | FCD type 2a | Superior temporal gyrus | SF | SF | 33 | 0.97 |
| 49 | M | 5 | 5 | Astrocytoma or glioneuronal tumor | Mesial (amygdala) | Glioneuronal tumor | - | SF | SF | 15 | 0.93 |
| 50 | F | 7 | 4 | Cystic leukomalacia | Anterior/polar | Glioneuronal tumor | - | SF | SF | 19 | 1.07 |
