# Supplementary material for: Extent of piriform cortex resection in children with temporal lobe epilepsy
Source: Ann Clin Transl Neurol. 2023 Jul 20;10(9):1613–22. doi: 10.1002/acn3.51852 (PMC10502684; doi:10.1002/acn3.51852)
Supplement: Supplementary file 2 — Appendix S1 [file ACN3-10-1613-s001.pdf]

PROTOCOL FOR:
Manual segmentation of the piriform cortex

Version 1.1; 5th July 2023

This protocol for manual segmentation of the piriform cortex is an adaptation of the protocol by Dr Marian Galovic et al[1] and the protocol by Dr Sabahat Iqbal et al[2]. These protocols are derived from the pathology-radiology validation study by Dr Gonçalves Pereira et al[3].

The following steps are taken:
1. Image acquisition
2. Image re-alignment
3. Contrast adjustment
4. Manual segmentation

STEP 1: IMAGE ACQUISITION

This manual segmentation protocol requires:
- MRI of the brain
- Without contrast
- 1mm isotropic resolution

The remainder of the segmentation protocol is not specific to a particular image analysis software, but requires software that allows manual markup per voxel. For the remainder of this written protocol, we have used ITKSnap to demonstrate the protocol: http://www.itksnap.org/

STEP 2: RE-ALIGNMENT

The protocol by Galovic et al measures the piriform cortex with the axial images aligned along the long-axis of the hippocampal body.

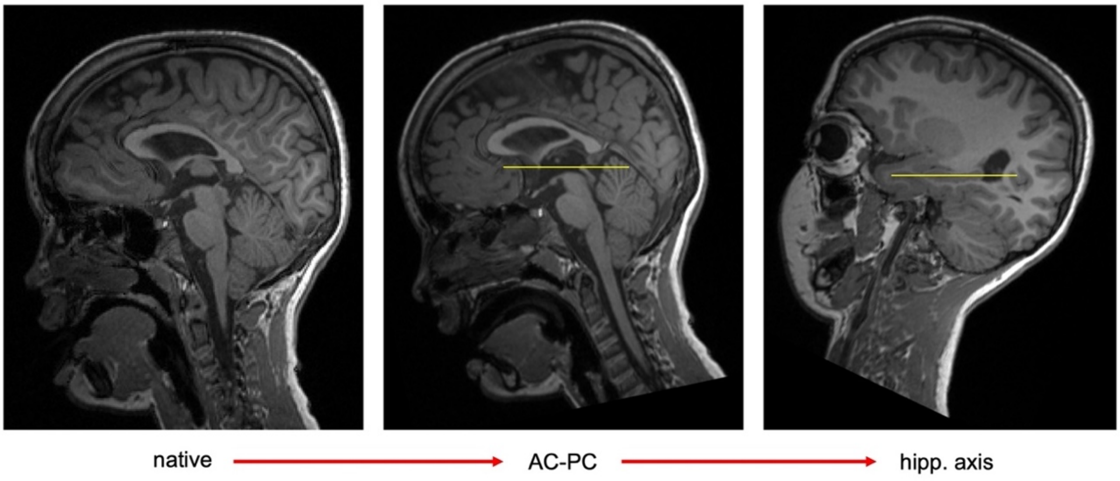

STEP 3: CONTRAST ADJUSTMENT

To ensure satisfactory identification of the piriform cortex and adjacent structures, the image contrast is adjusted. This is a patient-specific adjustment.

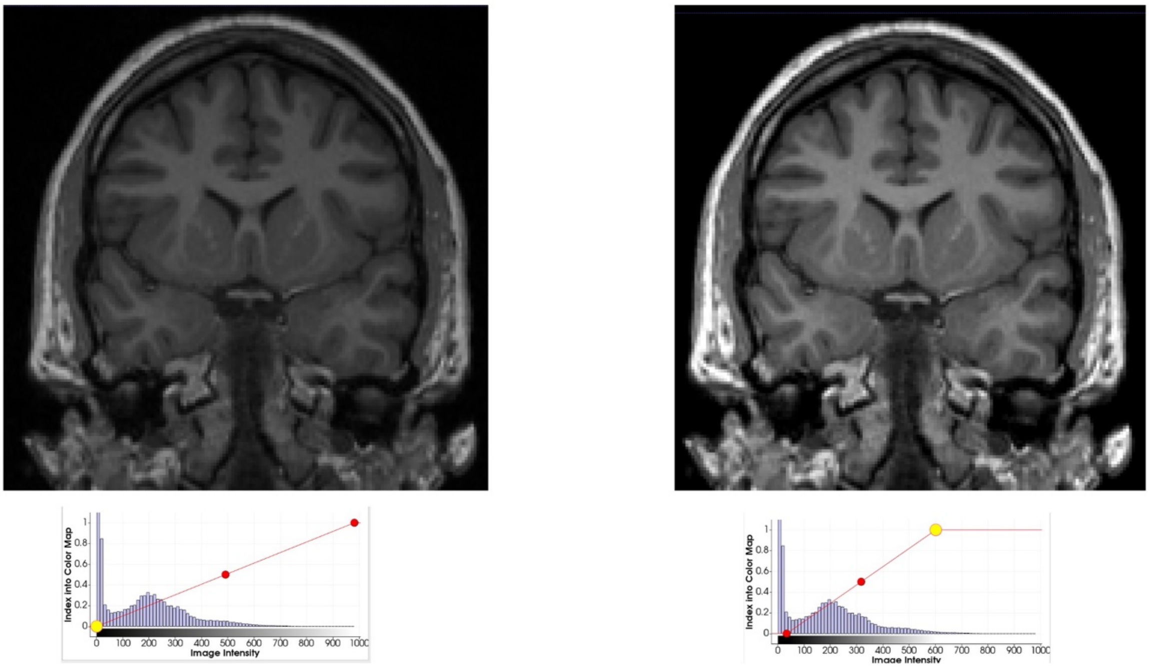

Compared to the image on the left, the image on the right shows an optimised image contrast profile.

STEP 4: MANUAL SEGMENTATION

This section describes each step for segmenting the piriform cortex.

Coronal sections are used throughout the segmentation protocol.

STEP 4A: Choosing the first slice

The first slice on which segmentation is performed is selected is the slice that first shows the appearance of the limen insulae. A bridge of white matter comes into view between the frontal and temporal white matter.

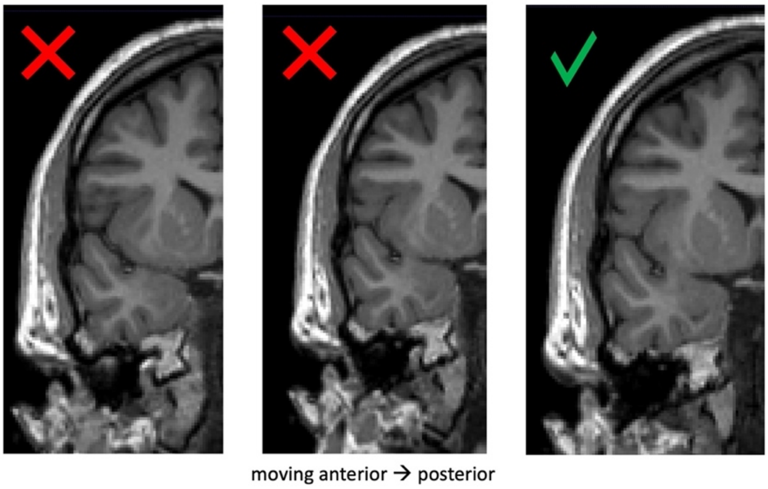

STEP 4B: Determining key landmarks on the first slice

Using the first slice determined by step 4B, four key landmarks are identified:

1. Depth of the 'endorhinal sulcus'
2. 'Apex' of gyrus semilunaris
3. Line of the olfactory sulcus
4. Medial limit of the frontal part of the piriform cortex, which is halfway between 1 and 3

The olfactory line is determined by following the olfactory sulcus posteriorly until it intersects the first slice.

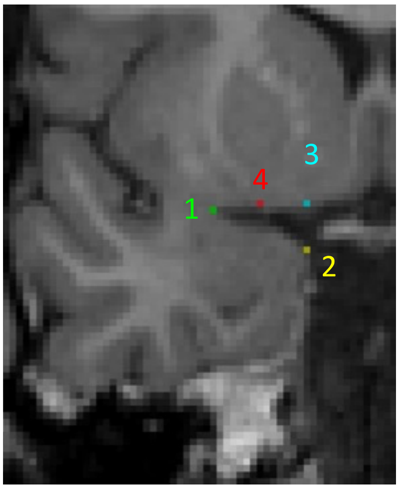

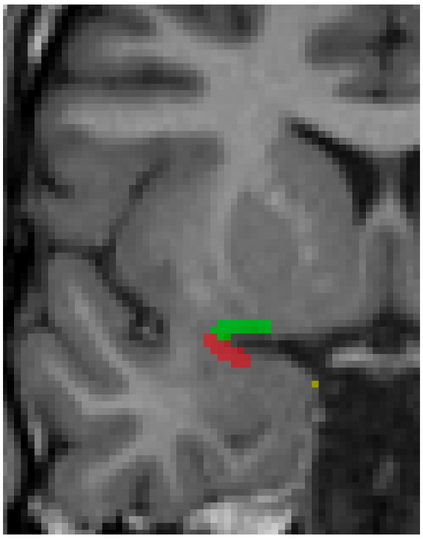

STEP 4C: Segmentation protocol for the first slice

The temporal part of the piriform cortex starts at the cortex at the depth of the endorhinal sulcus and stops at a distance 30% between the endorhinal sulcus and the apex of the gyrus semilunaris.

The frontal part of the piriform cortex starts at the cortex at the depth of the endorhinal sulcus and extends to the medial extent identified in step 4B.

As per the protocol by Galovic and Rojas, the depth of the piriform cortex is confined to 2mm (2 voxels in 1mm isotropic MRI).

STEP 4D: Segmentation protocol for the second slice

The second slice is the slice immediately posterior to the first. The third is the immediately posterior to the second, and so on in series.

The steps for the second slice in step 4D are similar to those in step 4C, but with the difference that the distance between the endorhinal sulcus and the apex of the gyrus semilunaris is changed to 50%.

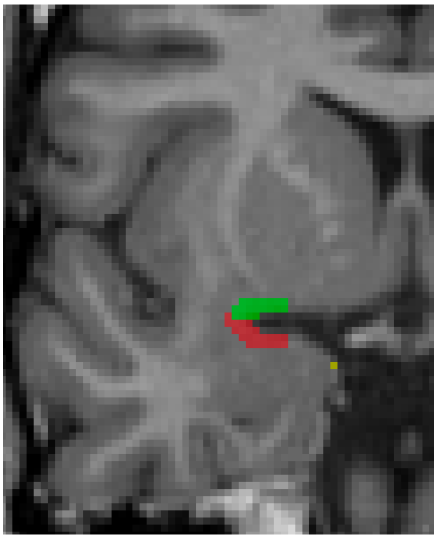

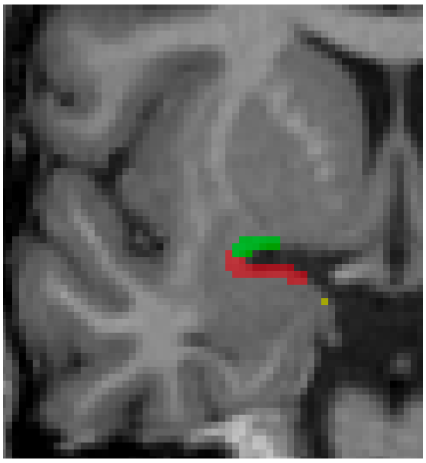

STEP 4E: Segmentation for the third slice

The steps for the second slice in step 4E are similar to those in step 4C and 4D, but with the difference that the distance between the endorhinal sulcus and the apex of the gyrus semilunaris is changed to 75%. If, however, the sulcus semiannularis (see step 4F) comes into view before the 75% mark, the segmentation stops at that point.

STEP 4F: Segmentation of the subsequent slices

In the subsequent posterior slices, the temporal part of the piriform cortex is segmented up until the sulcus semiannularis (SSA).

Moving posteriorly, the frontal part of the piriform cortex begins to narrow.

The final slice to be segmented is the slice before the mamillary bodies are convincingly seen.

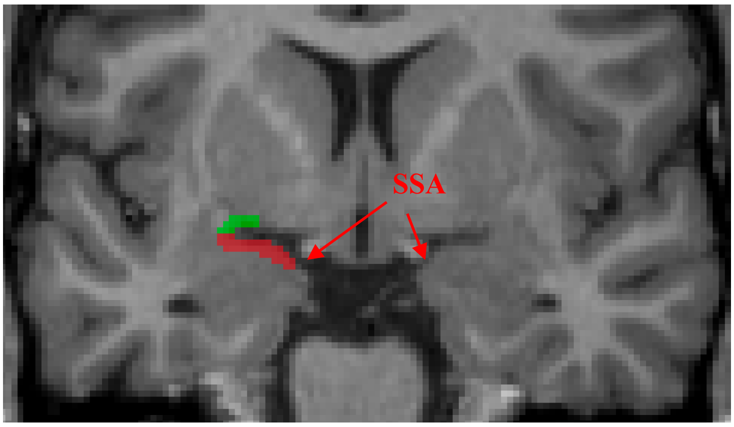

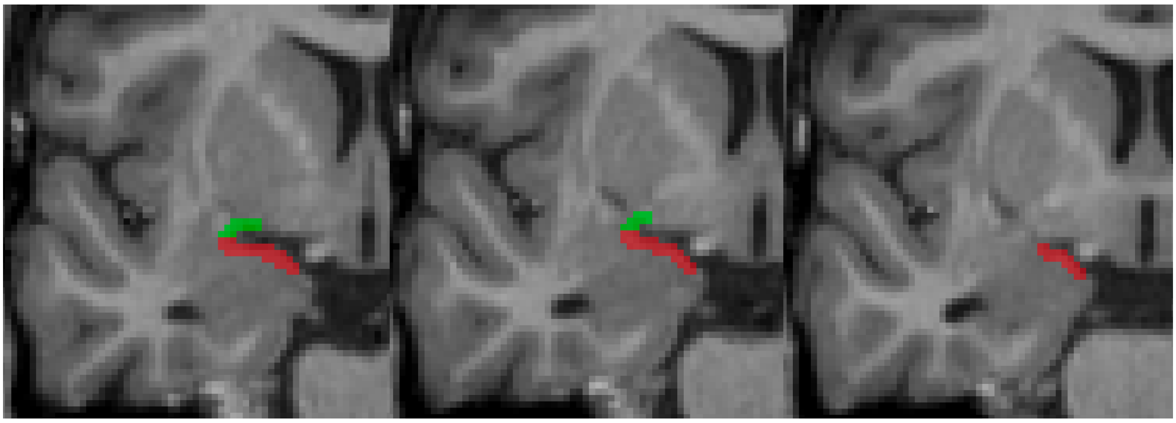

moving anterior → posterior

REFERENCES:

1. Galovic M, Baudracco I, Wright-Goff E, et al. Association of Piriform Cortex Resection With Surgical Outcomes in Patients With Temporal Lobe Epilepsy. JAMA Neurol. 2019;76(6):690.

2. Iqbal S, Leon-Rojas JE, Galovic M, et al. Volumetric analysis of the piriform cortex in temporal lobe epilepsy. Epilepsy Res. 2022;185:106971.

3. Gonçalves Pereira PM, Insausti R, Artacho-Pérula E, et al. MR volumetric analysis of the piriform cortex and cortical amygdala in drug-refractory temporal lobe epilepsy. AJNR. Am. J. Neuroradiol. 2005;26(2):319–32.
